# The Cost to the State of National Health Insurance

**Published:** 1915-06-19

**Authors:** 


### NATIONAL INSURANCE ACT DEVELOPMENTS.

### The Cost to the State of National Health Insurance.

When the National Health Insurance scheme was introduced, actuarial advice was obtained with the object of estimating the cost of the proposals then put forward, and to give an idea of the amount of the Parliamentary Funds that would have to be found for some years to come. The first report made by the actuaries was dated May 20, 1911, and this was followed by a revised report dated November 28 of the same year, which incorporated the revised estimates necessitated by additions to the scheme while it was under discussion in Parliament.

The recent issue of the Civil Service Estimates for the year ending March 31, 1916, presents a favourable opportunity for comparing the actual estimated expenditure with that anticipated three and a half years ago. The comparison is not, however, very readily accomplished, because in the original estimates allowance was only made for the State subsidy towards the benefits and the cost of their administration by the approved societies, and no account was taken of the cost of the central administration by the Insurance Commissioners.

As against this exclusion of the cost of the central administration from the original estimates there is necessarily, in the actual provision for the year, full allowance for all charges.

The estimates for the year are, however, given in great detail, and by extracting the appropriate figures from the accounts of the four separate Commissions and of the Joint Commission, it is possible to make a valid comparison with the figures which were put forward in 1911 as the sum likely to be incurred by the State in subsidising National Health Insurance during the year 1915-1916.

### Estimated Cost to the State for 1915-1916.

The figures for the current year's estimate are set forth in the following table:—

| | Central Administration. | Contributions, Benefits, etc. |
|---|---|---|
| | £ | £ |
| Joint Committee ... ... | 22,925 | 595,350 |
| English Commission ... | 293,056 | 4,607,300 |
| Welsh Commission ... ... | 40,253 | 317,300 |
| Scottish Commission ... | 61,027 | 658,000 |
| Irish Commission ... ... | 56,090 | 436,000 |
| Totals ... ... ... ... | 473,351 | 6,613,950 |

It is further to be noted that in addition to the above estimates provision is made under other departmental estimates, such, for instance, as office accommodation, stationery and printing, etc., for sums which in total amount to £393,017. Thus the total charge for the central administration amounts to no less than £866,368 for the year.

### Large Increase on the Original Estimate.

To return to the comparison with the original actuarial calculation, it will be found that the amount set forth as the expected State subsidy for the year 1915-1916 was £4,578,500, to which was to be added an uncertain sum on account of deposit contributors and married women voluntary contributors. Judging from the sample figures deduced by the actuaries as likely to be required to meet these two additional charges in other years (those for 1915-1916 are not given in their report), it is possible to place the original estimate at about £4,884,000.

Thus the actual cost of £6,613,950 is no less than £1,729,895 more than that originally anticipated. This excess is not primarily due to inaccuracy in the first estimates, though to some extent it is true that the expectation of sickness among women has been falsified by experience, but arises principally from subsequent additions to the benefits—particularly the medical and sanatorium benefits—and from the concessions granted to contributors falling into arrear with their payments on account of unemployment.

### The Total Cost.

The total cost to the State on account of National Health Insurance for the year is £7,480,318—or a matter of very nearly seven and a half million pounds—quite apart from the contributions payable by the insured persons themselves and their employers. Accounts do not yet show the amounts received in contributions, but it is probable that the total sum will not be much less than £18,500,000 for the year in question, and on this assumption the total cost to the nation for the National Insurance scheme is no less than £26,000,000. This is a large sum, being actually greater than the amounts voted annually by Parliament during recent years for the service of the National Debt. In view of this large expenditure it is incumbent upon all those who are working in any capacity for the health and well-being of the community to see that the funds are wisely administered, and although for the moment National Health Insurance does not occupy the same amount of attention as it did, it is nevertheless destined to play a foremost part in the restoration to health of our sailors and soldiers upon their return from the war.

